# Effect of ultrasound-guided pericapsular nerve group (PENG) block on pain during patient positioning for central nervous blockade in hip surgery: a randomized controlled trial

**DOI:** 10.1186/s12871-023-02245-3

**Published:** 2023-09-15

**Authors:** Hakan Aygun, Serkan Tulgar, Yavuz Yigit, Ayşe Tasdemir, Cengizhan Kurt, Caner Genc, Sezgin Bilgin, Nimet Senoğlu, Ersin Koksal

**Affiliations:** 1 Department of Anesthesiology, Bakircay University Faculty of Medicine Cigli Training and Research Hospital, Izmir, Turkey; 2 Department of Anesthesiology, Samsun University Faculty of Medicine, Samsun Training and Research Hospital, Samsun, Turkey; 3 Department of Emergency Medicine, Hamad Medical Corporation, Hamad General Hospital, Doha, Qatar; 4 Blizard Institute, Queen Mary University, London, United Kingdom; 5 Department of Orthopedic Surgery, Bakircay University Faculty of Medicine Cigli Training and Research Hospital, Izmir, Turkey; 6 Department of Anesthesiology, Ondokuz Mayıs University Faculty of Medicine, Samsun, Turkey

**Keywords:** Hip fractures, Patient positioning, Regional anesthesia, Pain management, Quality of recovery, Orthopedic anesthesia

## Abstract

**Background:**

Most patients with hip fractures are elderly patients with comorbidities, and well-managed pain management is associated with positive postoperative outcomes. In recent years, new indications for regional anesthesia techniques have been defined, and they have found more place in clinical practice. Herein we investigate the effect of US-guided PENG block on positioning pain and compare that effect to intravenous opioid in patients undergoing surgery under spinal anesthesia for hip fractures. Additionally, we sought to investigate the effect of PENG block on pain scores, opioid intake, time to first analgesic requirement, and quality of recovery within the first 24 h following surgery.

**Methods:**

In this study, patients were divided into the PENG (n = 42) and control group (n = 42) one hour prior to surgery. A team who was blinded to the assigned groups, collected and evaluated all data such as spinal anesthesia positioning pain, postoperative pain, opioid requirement.

**Results:**

Patients that underwent PENG had statistically significantly lower NRS scores after interventions, immediately before positioning, at positioning and at end of spinal anesthesia. Pain scores during positioning for spinal anesthesia were statistically significantly lower in the PENG group than in the control group (p < 0.001). Total morphine use over the first 24 h was extremely statistically significantly lower in the PENG group (p < 0.001).

**Conclusions:**

Positive outcomes of PENG block in patient positioning pain before spinal anesthesia, postoperative pain scores, and morphine consumption are consistent with similar studies. High patient satisfaction in patients who underwent PENG block contributes to the literature.

**Trial Registration:**

ClinicalTrials.gov Identifier: NCT04871061

## Introduction

Hip fracture surgery is a common orthopedic procedure, especially in elderly patients [1]. These fractures are quite common in adults aged 65 and older, and the one-year mortality rate is fairly high, ranging from 12–37% [2]. Recent studies have suggested that general anesthesia and spinal anesthesia are not superior to each other in patients undergoing hip fracture surgery [3, 4].

Untreated/poorly managed perioperative pain is directly linked to delirium, bad prognosis, and secondary chronic pain in hip fracture patients [5]. Therefore, when considering associated mortality, morbidity, and early recovery, the control of perioperative pain should be one of the anesthetist’s highest priorities, regardless of the anesthetic modality used.

Ultrasonography-guided (US-guided) regional anesthesia techniques are frequently used as part of a multimodal plan in the management of perioperative pain in patients with hip fractures. One of the most recently described blocks used in hip fracture surgery, US-guided pericapsular nerve group (PENG) block aims to directly block the articular branch of the femoral nerve, the articular branch of the obturator nerve and the accessory obturator nerve that selectively innervate the anterior aspect of the hip capsule [6]. Case reports and a limited number of clinical studies have reported PENG block as being effective in management of acute fracture-related pain, neuraxial anesthesia positioning pain, and postoperative pain in hip fracture patients [7].

Herein we investigate the effect of US-guided PENG block on positioning pain and compare that effect to intravenous opioid (fentanyl) in patients undergoing surgery under spinal anesthesia for a fractured hip. Additionally, we sought to investigate the effect of PENG block on pain scores, opioid intake, time to first analgesic requirement, and quality of recovery within the first 24 h following surgery.

## Materials and methods

### Study design

**Fig. 1:**
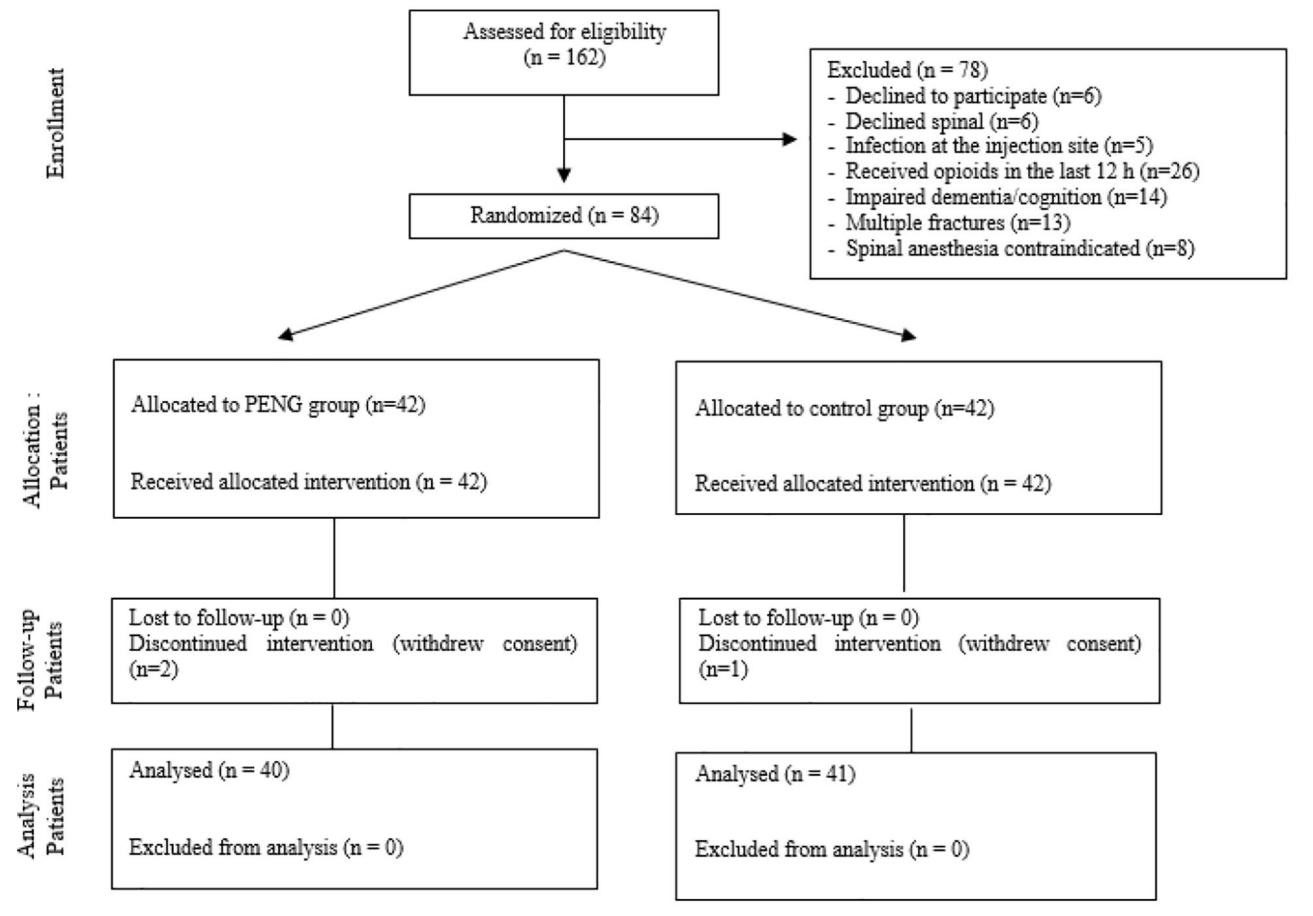
Flow chart of the study

Local Ethics Committee (Ondokuz Mayıs Universitesi - Klinik Araştırmalar Etik Kurulu : 2021/I740) and Ministry of Health approval as well as clinicaltrials.org registration (NCT04871061, Registration date: 04/05/2021) was obtained for this prospective, randomized, controlled, assessored study. The study was conducted in the Anesthesiology Department at Çiğli Training and Research Hospital from May 2021 to May 2022. The CONSORT checklist for the study can be found in Fig. 1. All participants gave written informed consent for participation in this study and the Declaration of Helsinki was adhered to.

Patients aged between 35 and 90 years that were scheduled to undergo hip fracture surgery under spinal anesthesia were recruited for the study. Those with contraindications for spinal anesthesia or PENG block (infection at injection site, low ejection fraction, coagulopathy etc.), dementia or similar cognitive function impairments that made participation in some components of the study inadequate, analgesia use within the last 12 h and those with another fracture or problem leading to chronic pain were excluded from the study. In addition, patients who were scheduled for urgent surgery due to hip fracture were excluded. Again, patients who could not obtain written informed consent to participate in the study were excluded from the study.

### Grouping and randomisation

Patients were separated into the PENG and control groups using the closed envelope method, one hour prior to surgery. Each patient was assigned a random ID that was used during all follow up and data collection. The same anesthesiologist performed randomisation and block in the PENG group (HA). However, this anesthesiologist was not involved in the evaluation of spinal anesthesia positioning pain evaluation or subsequent follow up.

All patients were taken into the block room before surgery and transferred to the operating room thereafter. Evaluators who were blinded about the interventions and groups were taken to the room ‘just before the spinal anesthesia positioning’. This blinded assesor team (AT, CK) collected and evaluated all data such as spinal anesthesia positioning pain, postoperative pain, and opioid requirement.

### Interventions

**Fig. 2:**
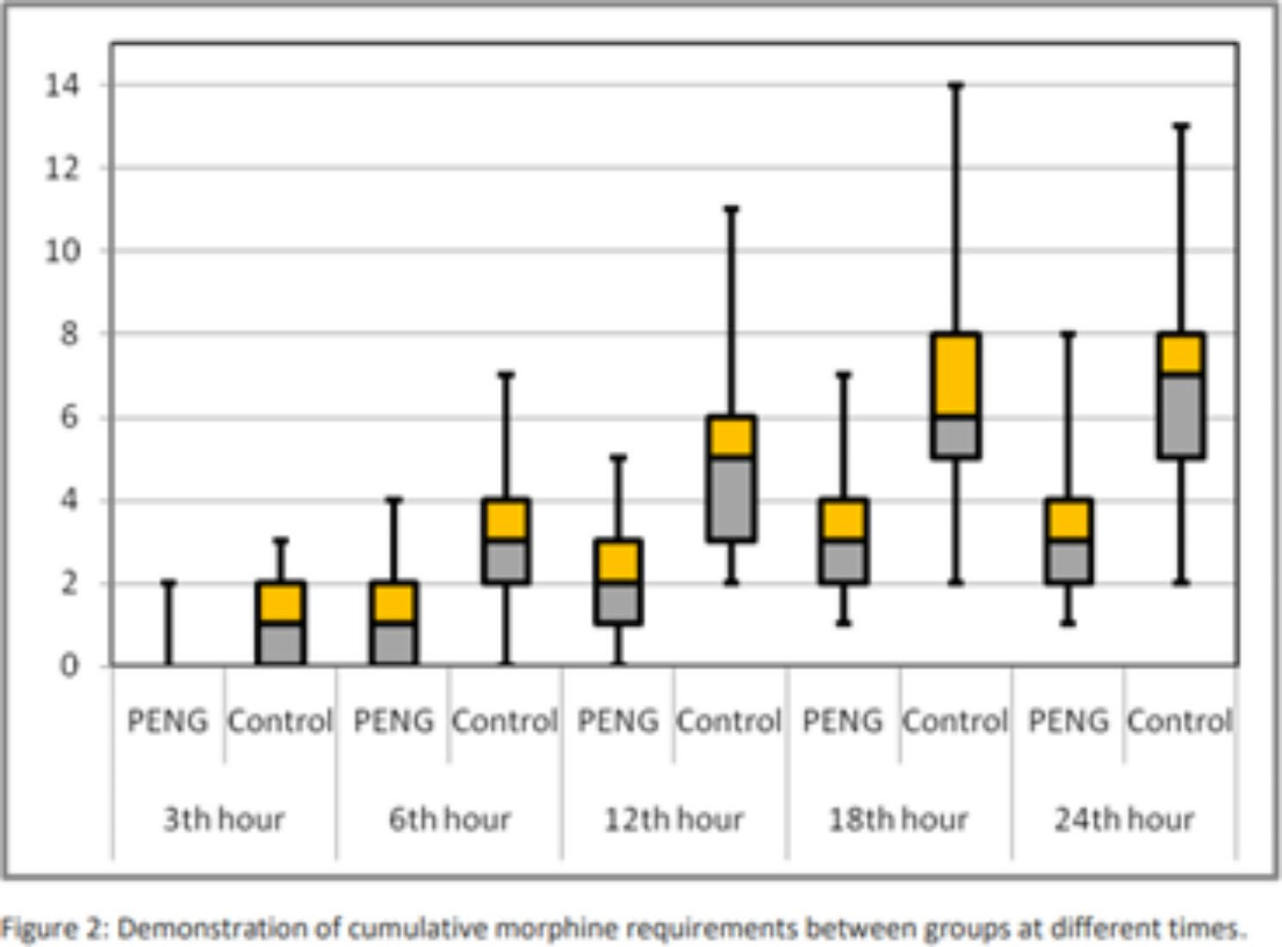
Demonstration of cumulative morphine requirements between groups at different times

In the control group: 1.5 mcg/kg of IV fentanyl was performed five minutes prior to spinal anesthesia. For those in the block group, PENG was administered 20 m prior to spinal anesthesia. All blocks were performed by the same anesthesiologist in the block room. Patients underwent basic monitoring and O_2_ was administered via nasal cannula. Vascular access was obtained via the non-dominant hand. After appropriate skin antisepsis, all blocks were conducted with the in-plane technique, utilizing a 10 cm, 21G block needle using a convex USG transducer on the side of the hip fracture. The USG transducer was placed on the anterior superior spina iliaca in the transverse plane. Thereafter, the transducer was slowly shifted caudally to identify the spina iliaca anterior inferior, with the transducer positioned slightly obliquely, superolaterally and inferomedially. The transducer was positioned so that the femoral artery, femoral nerve, iliacus muscle, psoas tendon, iliopubic eminence and spina iliaca anterior inferior structures were all visible within the field of view. Particular attention was paid to avoid having the hip joint and femoral head within the field of view. The needle was advanced in-plane from lateral to medial until the needle tip was placed between the iliopsoas tendon (IPT) and periosteum, lateral to iliopubic eminence (IPE). First; 1–2mL of saline solution was injected to observe the spread under the iliopsoas muscle (IPM) with the IPT lifted up, then 20 mL of 0.25% bupivacaine was administered (Fig. 2) [8]. Negative blood aspiration was performed following every 5 mL of injection.

## Spinal anesthesia management

All patients received spinal anesthesia in a seated position under sterile conditions. A 25 G spinal needle was inserted via L3-L4 or L4-L5 interspinous space and 10-12.5 mg of bupivacaine heavy was administered. No additives, including intrathecal morphine, were utilized. The patients were immediately placed on their backs following the intrathecal injection, and the side to be operated on was held at a 30 degree angle for 5 min. Surgery was commenced following confirmation of successful spinal anesthesia using a pinprick test.

### Pain evaluation, postoperative analgesia and quality evaluations

The Numeric Rating Scale (NRS) was utilized to assess pain intensity. NRS, a unidimensional measurement of adult pain severity, is a segmented numeric version of the visual analog scale (VAS) in which a respondent selects a whole number (ranging from 0 to 10) that best depicts the degree of his/her pain. The 11-point numeric scale spans from ‘0’ for one pain extreme (e.g., “no pain”) to ‘10’ for the opposite pain extreme (e.g. “pain as bad as you can imagine” or “worst pain imaginable”).

Time frames were defined as follows:

- Preoperatif NRS: NRS at rest before intervention (PENG or iv fentanyl).
- Prepositioning NRS: NRS score at rest immediately before positioning.
- Positioning NRS: NRS at positioning, 5 m after intervention in control group or 20 m after intervention in PENG group.
- Post-positioning NRS: NRS at rest following spinal anesthesia in the supine position.

NRS was also measured, at rest, at the 3rd, 6th, 12th, 18th and 24th hours after the end of surgery.

All patients received 1 g of iv paracetamol immediately following the end of surgery in the recovery room and every 6 h thereafter in the first 24 h, postoperatively. The patient-controlled analgesia (PCA) device was commenced intravenously in all patients in the recovery room. Patients were instructed to self-administer pain relief by pressing the PCA device when NRS ≥ 4/10. The PCA consisted of 0.5 mg/mL of morphine that was delivered without a basal infusion, followed by boluses of 1 mg each and a 20-minute lockout after each administration. Patient follow-up lasted for 24 h, PCA device was removed after 24 h.

### Outcomes measurements

Primary outcomes were positioning pain scores - the maximum reported NRS score at 5 min after intervention in the control group and 20 min after intervention in the PENG group. Secondary outcomes were:

1. Duration of spinal anesthesia performance: measured in minutes, from the start of positioning maneuvers to removal of spinal needle.
2. Quality of patient’s position: evaluation of patient position by anesthesiologist that performed spinal anesthesia as “unsatisfactory”, “satisfactory”, “good” or “very good” (0-1-2-3 points, respectively).
3. Analgesic consumption: Daily morphine consumption from PCA.

In addition, parameters such as NRS scores, first analgesia requirement time, etc. were also evaluated in the above-mentioned time frames. At the postoperative 24th hour, the Quality of Recovery-15 (QoR-15) questionnaire was completed to determine the quality of recovery.

### Sample size and statistical analysis

A previously performed pilot study of 10 patients revealed the maximum NRS score when positioning for spinal anesthesia to be 4.2 ± 1.30 vs. 3 ± 1.22 in the control and PENG groups, respectively. This data was utilized using an alpha 5%, beta 10% and power of 95% to calculate a minimum sample size of 29 patients per group. Taking into consideration possible drop outs, loss to follow up and secondary outcomes, the decision was taken to have each group include 42 patients.

Statistical evaluation of data was conducted with SPSS for Windows, Version 16.0. (SPSS Inc, Chicago, USA). Normal distribution was evaluated through the Kolmogorov-Smirnov test. Continuous variables were expressed as mean ± standard deviation, and median (25th–75th percentiles). Continuous variables of equal variance were analyzed using the t-test and non-normally distributed data with the Mann Whitney U test. Chi-square was used for comparison of ratios and categorical data was compared using Fisher’s exact test. Finally, Kaplan-Meier analysis and Wilcoxon test was used when comparing time to first analgesia requirement. p < 0.05 was accepted as being statistically significant in all data except for postoperative NRS scores, where after Bonferroni correction statistical significance was considered when p < 0.01.

## Results

One hundred and sixty two patients were assessed for eligibility. Following the exclusion of 78 patients, a total of 84 patients were randomized into two groups. The CONSORT Flow diagram is shown in Fig. 1. Final analysis was performed with 40 patients in the PENG group and 41 in the control group, respectively. In the PENG group, 21 patients underwent partial hip hemiarthroplasty, 19 patients received proximal femoral nailing (PFN). In the control group, 20 patients underwent partial hip hemiarthroplasty, and 21 patients had PFN (Table 1). And groups were similar in terms of types of surgery (p > 0.05).

**Table 1:** Comparison of demographic data and first opioid requirement between groups. Surgery types are expressed as number of patients whereas other data is expressed as mean ± standard deviation

	PENG Group (n:40)	Control Group (n:41)	p
	Mean ± SD	Mean ± SD	
Age (years)	73.28 ± 9.54	73.26 ± 7.62	0.991
Gender F/M (n)	26/14	29/12	0.580
Weight (kg)	72.57 ± 13.28	68.17 ± 9.06	0.084
Height (m)	1.63 ± 0.09	1.61 ± 0.08	0.598
BMI (Body Mass Index)	27.14 ± 4.23	26.05 ± 3.14	0.191
Type of Surgery (Hip hemiarthroplasty / Proximal femoral nailing)	21/19	20/21	
Spinal Anesthesia Performance Time (min)	4.56 ± 0.43	5.28 ± 0.44	***< 0.0001***
Surgery Time (min)	79.57 ± 8.18	78.65 ± 8.85	0.628
Time of First Opioid Requirement (hours)	9.12 ± 3.61	5.07 ± 2.55	***< 0.001***

The demographic characteristics of participants are demonstrated in Table 1. These characteristics were similar between groups. Performance time of spinal anesthesia was statistically significantly longer in the control group (4.56 ± 0.43 vs. 5.24 ± 0.44, respectively, p < 0.001).

Preoperative NRS scores were similar between two groups (p = 0.0885). As the primary outcome of the study; patients that underwent PENG had statistically significantly lower NRS scores after interventions, immediately before positioning, at positioning and at end of spinal anesthesia (p < 0.0001, p < 0.0001 and, p < 0.0001, respectively). Additionally, the anesthesiologist evaluated the quality of the patient’s position, and it was statistically significantly higher in the PENG group when compared to the control group (p < 0.0001) (Table 2).

**Table 2:** Comparison of peri-positioning NRS scales, quality of patient’s position. Data are expressed as median (percentiles 25–75)

	PENG Group	Control Group	p
Preop NRS	5 (4-5.25)	5 (4–6)	0.885
Prepositioning NRS	2 (2–2)	4 (3–4)	***< 0.0001***
Positioning NRS	3 (3–4)	5 (4–5)	***< 0.0001***
Post positioning NRS	2 (2–2)	3 (2–3)	***< 0.0001***
Quality of patient’s position	3 (3–3)	2 (2–2)	***< 0.0001***

Table 3 shows the cumulative consumption of morphine at 3th, 6th, 12th, 18th, 24th hours and NRS scores throughout time, as well as the average time until first PCA use. In the PENG group, there was a significant reduction in the cumulative morphine requirement at the 24th hour compared to the control group (3.13 ± 1.716 mg vs. 68 ± 2.72 mg, p < 0.001). Furthermore, the PENG group consistently demonstrated lower cumulative morphine consumption across all time frames compared to the control group (p < 0.001). Figure 2 presents a box plot graph illustrating the cumulative opioid requirements across all time frames.

**Table 3:** NRS scores, total morphine consumption, time to first PCA use and QoR 15 scores for groups. Data are expressed as median (percentiles 25–75)

	PENG Group (n:40)	Control Group (n:41)	p
Hours	**NRS**		
3rd	0 (0–3)	3 (3–4)	***< 0,001***
6th	3 (3–3)	4 (4–5)	***< 0,001***
12th	3 (3–4)	5 (4–5)	***< 0,001***
18th	3 (3–3)	3 (3–3)	0.375
24th	2 (0–2)	2 (0–2)	0.177
Hours	**Total Morphine Requirement (mg)**		
3rd	0 (0–0)	1 (0–2)	***< 0.001***
6th	1 (0–2)	3 (2–4)	***< 0.001***
12th	2 (1–3)	5 (3–6)	***< 0.001***
18th	3 (2–4)	6 (5–8)	***< 0.001***
24th	3 (2–4)	7 (5–8)	***< 0.001***
	**Time to First PCA demand (h)**		
	8 (6.75-12)	4 (3–7)	***< 0.001***
	**Quality of Recovery 15 (QoR 15) score**		
	111.02 ± 9.67	99.51 ± 9.45	***< 0.001***

Requirement of first analgesia was later in the PENG group (9.12 ± 3.61 h vs. 5.07 ± 2.55 h, p < 0.001). NRS scores were significantly lower at 3rd, 6th and 12th hours in the PENG group, but similar at other times. According to the QoR 15 scores, which is a questionnaire used to measure the quality of recovery in patients, the PENG group demonstrated significantly better recovery quality compared to the control group (111.02 ± 9.67 vs. 99.51 ± 9.45, respectively, p < 0.001).

There were no complications that we detected or reported afterwards, either in the application of PENG block or in the application of spinal anesthesia.

## Discussion

Herein we have demonstrated that application of a PENG block prior to spinal anesthesia in patients undergoing surgery for hip fracture not only reduces pain associated with spinal anesthesia positioning, but also increases practitioner satisfaction and decreases postoperative analgesic consumption compared to a control group. In addition, QoR 15 scores were found to be greater for patients that underwent PENG block.

Current guidelines recommend fascia iliaca block or local anesthetic infiltration in hip fracture surgeries, where pain is highly expected [9]. Control of postoperative pain is of vital importance, especially in elderly and fragile patients undergoing major orthopedic surgery. PENG block has previously been defined for use in the management of acute and perioperative pain observed in hip fracture surgery [7, 10, 11].

In a prospective mini-cohort investigating the effects of PENG block in patients undergoing hip surgery, PENG was applied to 20 consecutive patients undergoing hip surgery with satisfactory results reported with regard to spinal anesthesia positioning [12]. However, the study lacked a control group and the authors did not report the effect of the block postoperative period, reducing the value of the study. In a separate controlled study evaluating effects of PENG block on spinal anesthesia positioning in patients undergoing surgery for hip fracture [13], authors reported that patients who underwent PENG block had less positioning-related pain when compared to patients who were positioned directly without any intervention, and that practitioner satisfaction was considerably higher (90%) compared to the control group (40%). However, postoperative pain and analgesia requirements were not evaluated in this study. Ideally, while investigating the effectiveness of the PENG block, an intervention/medication should be administered to the control group before positioning so that no patient group is condemned to pain. We therefore applied intravenous fentanyl to the control group in our study. Diakomi et al. [14] investigated the effect of fascia iliaca compartment block on positioning in neuraxial anesthesia and similarly to our study used 1.5 mcg/kg iv fentanyl. The authors reported that fascia iliaca block both reduces pain during patient positioning, delays the first analgesia requirement in the postoperative period and reduces 24-hour opioid requirement [11], similar to our results. Among the hypotheses put forward regarding the various mechanisms of action of PENG was the possibility that a high volume PENG block would have a comparable effect to a fascia iliaca/lumbar plexus block [15]. However, an anatomical study does not support this and it is stated that local anesthetic diffusion cannot capture the obturator nerve and its branches even if the volume is increased [16].

In a study where the effect of PENG block in hip fracture surgery was compared to femoral block, the authors aimed to evaluate the postoperative analgesic effects and quadriceps weakness in both blocks [17]. This study received plenty of methodological criticism as patients undergoing both spinal and general anesthesia were included and patients in the control group underwent femoral block [18].

Our study is the first to investigate the effects of PENG block on both spinal anesthesia positioning and postoperative analgesic efficiency and QoR 15 score. Undoubtedly, additional comparative research on the use of PENG block in hip surgery is required.

Although some publications hypothesize that lumbar plexus-like effects can be seen in PENG block when using 30 mL or more of LA, such an effect is not expected with 20 mL. As a result, it is obvious that PENG block will be insufficient for cutaneous anesthesia. Therefore, better results may be obtained with the combination of PENG block with other blocks in patients undergoing hip fracture surgery. A volunteer study on the cutaneous innervation of hip surgeries reported that subcostal nerve block and transversalis fascia plane block could be effective [19]. In order to achieve more effective results, new combinations - in light of comprehensive anatomical studies, will be needed.

The majority of patients undergoing surgery for hip fracture have a significant degree of fragility. This places patients at an elevated risk for LAST and compels clinicians to exercise greater caution. Volume and concentration of local anesthetic should be given careful consideration. In this investigation, we utilized 20 cc of local anesthetic for PENG block without experiencing any complications.

Our study has some limitations. First of all, we did not assess quadriceps weakness in patients, which is crucial for early recovery. However, we did not observe any long-term consequences linked to quadriceps weakness. Secondly, we could have compared the efficacy of PENG block with a technique such as fascia iliaca block, which has previously been demonstrated to improve positioning in clinical studies. However such a design would mean we would have been unable to determine the long-term effects of PENG block by comparing it to a control group. Another limitation was that some patients in the control group had relatively high NRS scores, although all NRS scores were in the acceptable “bearable pain” category. Spinal anesthesia performance time is one of our outcomes, but it can also be affected by many factors of the patient such as anatomy, obesity, and other factors such as the experience of the practitioner.

## Conclusions

Our study has shown that preoperative PENG block is effective both in eliminating the pain associated with spinal anesthesia positioning, and in reducing the need for opioids in the postoperative period in patients undergoing hip fracture surgery. In addition, we have demonstrated that PENG block increases Quality of Recovery (QoR 15) score in these patients.

### What is known

PENG block in hip surgeries is associated with positive postoperative results.

PENG block reduces the pain scores and morphine consumption postoperatively.

### What is new

PENG block significantly reduces pain scores during positioning for spinal anesthesia in patients with hip fractures.

PENG block significantly increases Quality of Recovery 15 (QoR 15) scores during positioning for spinal anesthesia in patients with hip fractures.

## Data Availability

The datasets used and/or analysed during the current study available from the corresponding author on reasonable request.
